# Mitral annular disjunction and mitral valve prolapse: echocardiographic-MRI insights into cardiac function

**DOI:** 10.1007/s10554-026-03709-6

**Published:** 2026-04-27

**Authors:** Max D. King, Andrew C. Peters, Stuart Karimi, Alyson N. Owen, Baskaran Sundaram, Behzad B. Pavri

**Affiliations:** 1 Hospital of the University of Pennsylvania, 3400 Spruce St Philadelphia, 19104 USA; 2 Sidney Kimmel Medical College, 1025 Walnut St, Philadelphia, 19107 USA; 3 Thomas Jefferson University Hospital Department of Cardiology, 1025 Walnut St, Philadelphia, 19107 USA

**Keywords:** Mitral valve prolapse, Mitral annular disjunction, Cardiac magnetic resonance imaging, Cardiac output, Cardiac index, Myocardial fibrosis

## Abstract

*Background*: Mitral annular disjunction (MAD) may accompany mitral valve prolapse (MVP) and is linked to ventricular arrhythmias. Its impact on global cardiac performance (specifically cardiac index (CI) and output (CO)), remains incompletely defined. Cardiac MRI (CMR) allows precise measurement of MAD, assessment of mitral annular dynamics, and detection of myocardial fibrosis via late gadolinium enhancement (LGE). We investigated both CMR and TTE metrics in patients with varying degrees of MAD compared to MVP-only. *Methods and results*: Fifty-six patients who underwent CMR and TTE (mean age 61, 33 female) were divided into MAD ≥2 mm and MVP (the “MAD + MVP” group, n = 41) or MVP-only (n = 15). MAD was classified as <5 mm, ≥5 mm, and ≥ 10 mm in peak systole after excluding pseudo-MAD. LV volume, stroke volume, ejection fraction, cardiac output (CO), cardiac index (CI), mitral regurgitation (MR), and LGE were assessed. Any MAD was associated with lower CI versus MVP-only (p = 0.03). MAD ≥5 mm and MAD ≥ 10 mm showed reduced CO (p = 0.04 and 0.04, respectively) and CI (p = 0.04 and 0.03, respectively) versus MVP-only. LGE was more frequent in MAD ≥ 10 mm than in MAD < 10 mm (p = 0.028). MR incidence, volume, or fraction did not differ across groups. TTE showed agreement with CMR in MAD detection and quantification (p = 0.269), with minimal inter-observer variability (p < 0.001). *Conclusion*: MAD is associated with impaired cardiac performance independent of MR and with increased myocardial fibrosis in severe cases. These findings suggest heightened electromechanical stress in MAD compared to MVP-only, which needs to be clinically correlated. TTE reliably detects and quantifies MAD, supporting its use alongside CMR for assessment of MAD.

**Supplementary Information:**

The online version contains supplementary material available at 10.1007/s10554-026-03709-6.

## Introduction

MAD is increasingly recognized as a distinct structural abnormality, although often associated with mitral valve prolapse (MVP) [1, 2]. It is characterized by macroscopic detachment of the mitral annulus supporting a portion of the posterior mitral leaflet from the adjacent ventricular myocardium. Hence, MAD is associated with the partial loss of mechanical annular function linked to the loss of normal annulo-ventricular anchoring while electrical isolation of the left atrium/ventricle is maintained. MAD predominantly affects the insertion of the posterior leaflet, extending laterally variably under all scallops but preferentially under the P2 scallop [3–5]. The anterior (aortic) leaflet is usually not disjunctional due to the aortomitral continuity.

During ventricular systole, the posterior leaflet of the mitral valve slides upwards and away from the mitral annulus due to non-anchoring, and away from the papillary muscles to which it remains tethered by the chordae tendinae [6]. The repeated abnormal traction may impose mechanical stress on the papillary muscles and the inferobasal left ventricular wall [7], leading to fibrotic remodelling in these regions. Such myocardial fibrosis and altered mechanics have been implicated as substrates for adverse ventricular remodelling and as potential niduses of electrical instability [8]. 

Advances in imaging, particularly Cardiac MRI (CMR) have significantly improved detection and characterization of MAD. CMR offers high spatial resolution capable of identifying disjunction as small as 1 mm [9]. Further, CMR offers unique advantages including precise volumetric quantification, mitral annulus measurements throughout the cardiac cycle, and appraisal of scar burden by late gadolinium enhancement (LGE) [4]. These capabilities make CMR a valuable modality for understanding the mechanical and structural consequences of MAD . While transthoracic echocardiography (TTE) is the most common initial test to detect MAD [10], its sensitivity for detecting MAD is limited and requires a meticulous “frame-by-frame analysis” [11] of the heart in systole, making it easily overlooked if not explicitly sought. 

The clinical significance of MAD has been most robustly linked to arrhythmic risk, with several studies demonstrating an association between MAD, ventricular arrhythmias, and sudden cardiac death in select MVP phenotypes [12, 13]. However, the functional impact of MAD on global cardiac performance, specifically on cardiac index (CI) and cardiac output (CO), remains incompletely defined. Indeed, recent reviews emphasize the need for further research to elucidate the hemodynamic consequences of MAD, including its potential impact on cardiac index and output [14]. 

### Aims of this study

To determine the impact of MAD and MVP on cardiac function by MRI and to evaluate the reliability of TTE for MAD detection and quantification.

## Methods

### Establishing groups

We developed an IRB-approved registry for a retrospective analysis at Thomas Jefferson University Hospital of patients who underwent CMR from 2019 to 2023, identifying cases of MAD and/or MVP based on data extracted from CMR and TTE reports. Patients with prior mitral valve surgery were excluded.

**Fig. 1:**
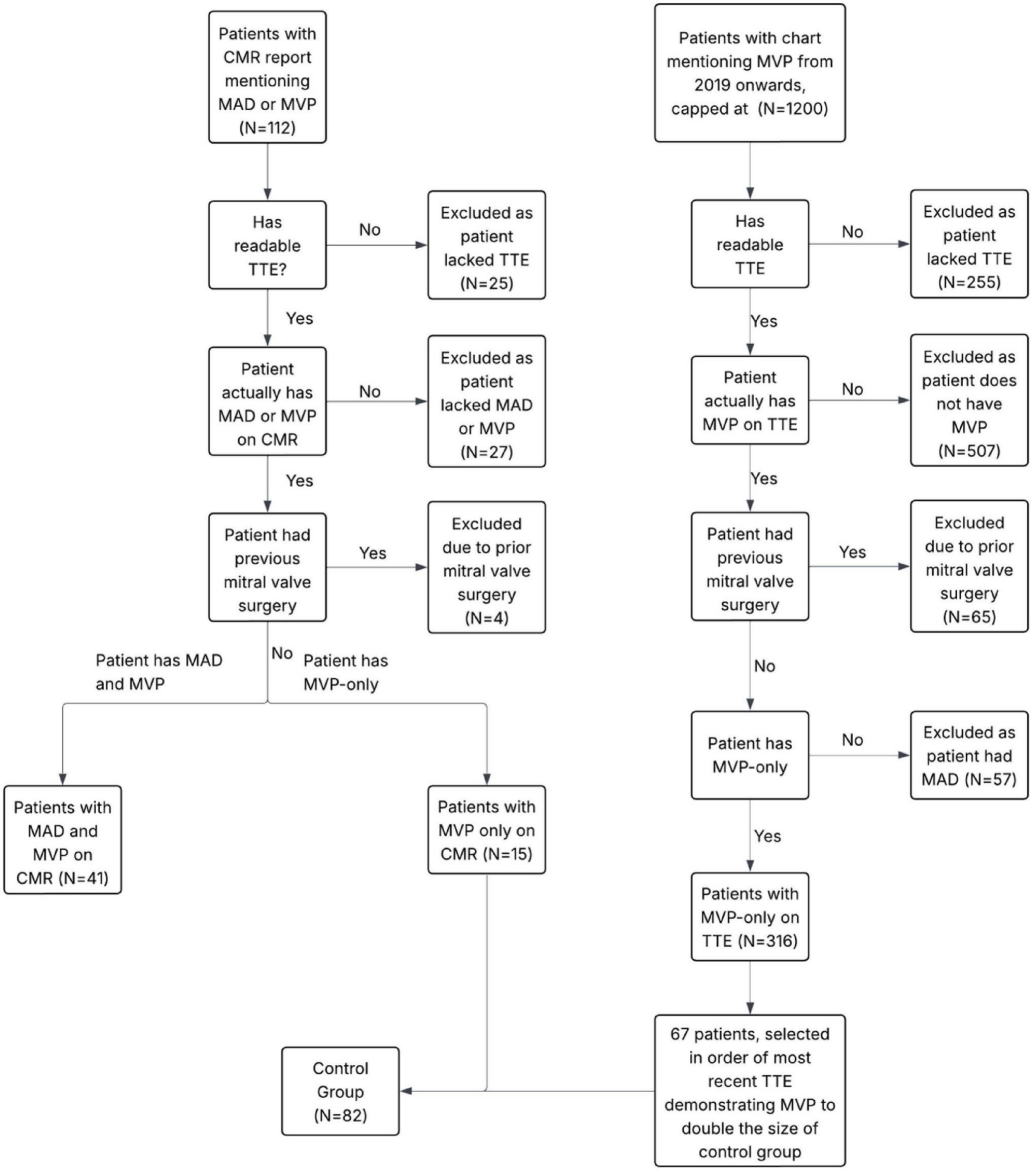
Flowchart detailing selection of patients into two groups: those with MAD and those with MVP-only (control)

This CMR registry identified two groups: patients with any degree of MAD and MVP (referred to as the *MAD + MVP* group) and patients with *MVP-only*. CMR imaging was used to precisely quantify MAD extent, as TTE has limited resolution when measuring small amounts of disjunction. From the CMR database, the *MVP-only* group comprised a small number of patients; hence, for echocardiographic comparisons, we also reviewed our echocardiography database for all patients diagnosed with MVP from 2019 to 2023. Patients meeting the same inclusion criteria were added to the *MVP-only* control group based on the most recent TTE demonstrating MVP *without* MAD (each TTE was carefully reviewed to confirm the absence of MAD) until the *MVP-only* group was double that of the *MAD + MVP* group. Thus, the final two groups were as follows (Fig. 1).

- Patients with MVP and MAD confirmed via CMR (referred to as the *MAD + MVP* group).
- Patients with *MVP-only* confirmed by either CMR and TTE or TTE alone.

Further, patients in the *MAD + MVP* group were separated into groups of <5 mm, ≥5 mm, and ≥10 mm by measurement on CMR.

### Data collection

**Fig. 2:**
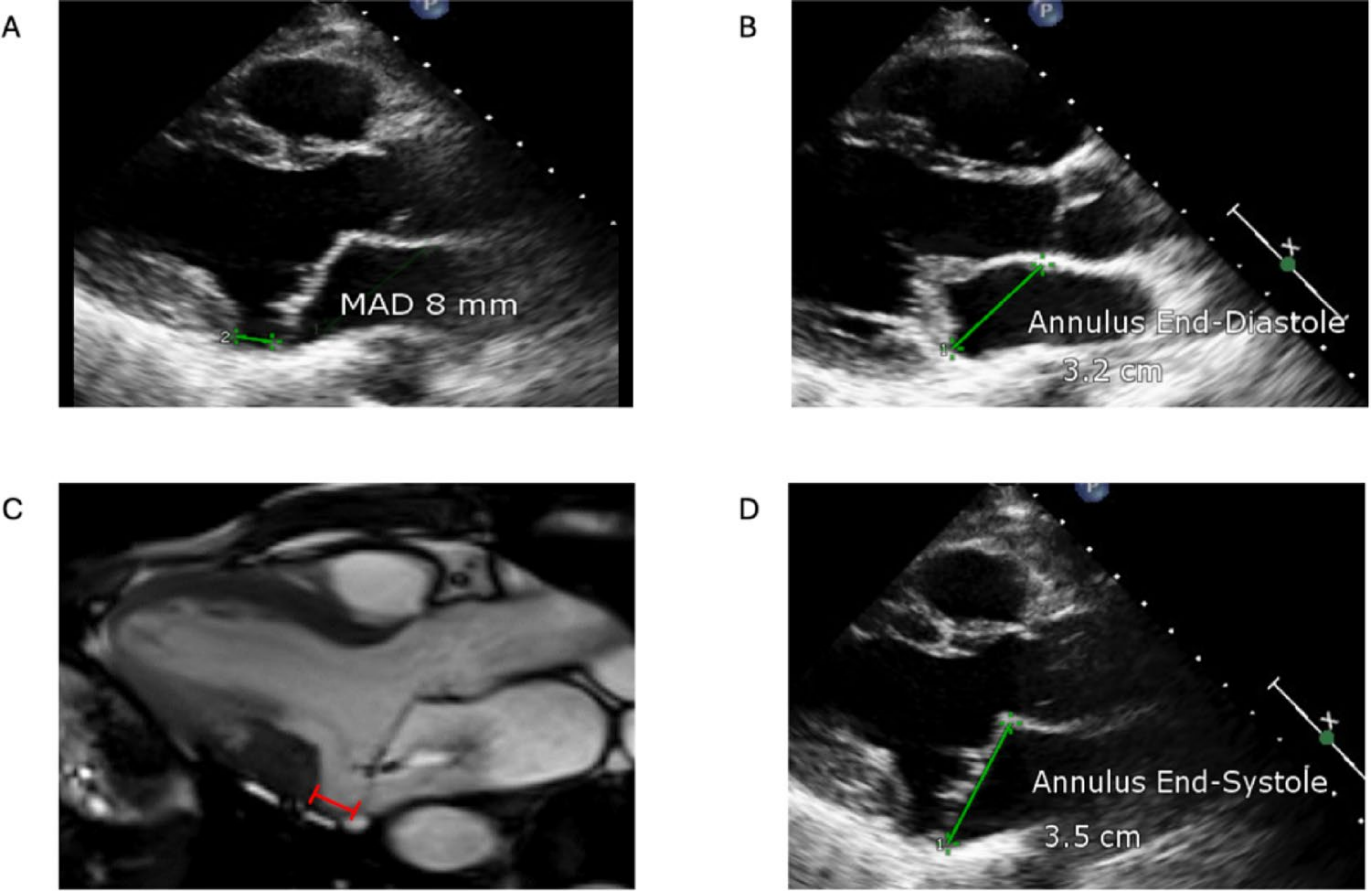
A and C illustrate MAD measurement via TTE in parasternal long-axis view and CMR measurement of MAD, respectively. B and D illustrate the measurement of the mitral annulus in diastole and systole in the parasternal long-axis view on TTE, respectively

Patient’s TTEs were selected as the nearest subsequent TTE by date after their CMR, or, if there was no CMR, the most recent TTE was selected. On TTE, MAD was measured in the parasternal long-axis view as the distance from the mitral annulus to the posterior leaflet (Fig. 2) at end-systole. Measurements were obtained from three cardiac cycles and averaged. The mitral annulus was measured at end-systole and end-diastole (Fig. 2), and the presence of the Pickelhaube sign on tissue Doppler imaging of the mitral annulus was recorded in the apical views. Additionally, patients were excluded if “pseudo-MAD” (when the posterior mitral leaflet flattens against the atrial wall during ventricular systole) [15] was recognized. We also collected data on the presence of mitral leaflet prolapse, severity of prolapse, and description and severity of mitral regurgitation (MR). Inter-reader variability ofTTE interpretation was assessed in ten subjects.

Cardiac MRIs were performed on 1.5 T GE scanners. CMRs were assessed by experienced cardiothoracic radiologists for the presence of MAD length ≥2 mm (Fig. 2) and MVP height using cine-MRI steady state free precession (SSFP) images in the 3-chamber view at peak systole, after excluding pseudo-MAD. The maximal extent of disjunction was recorded.

LV volumes and ejection fraction were derived from a gated short-axis cine (SSFP) stack with 8 mm slice thickness and an 8 mm interslice gap, with post-processing performed using Vitrea software. Cardiac output and index were assessed using in-plane phase-contrast imaging with a velocity encoding (VENC) of 150 cm/s. Mitral valve regurgitant volume was calculated as LV stroke volume (by volumetry) - aortic stroke volume (by phase contrast). Regurgitant fraction was calculated as (LV stroke volume - aortic stroke volume) / LV stroke volume. LGE was evaluated using phase-sensitive inversion-recovery sequences acquired across multiple imaging planes.

### Statistical analysis

Statistical analysis was performed using Welch’s unpaired T test for continuous variables and the Chi-square test for categorical variables (Fisher’s exact test was used for expected cell counts < 5). ANOVA analyses were performed when comparing data across the MAD subgroups described above. P-values < 0.05 were considered significant.

We also compared *MVP-only* patients detected by CMR and TTE to those detected by TTE alone to ensure no statistically significant differences when forming the combined control group. Further, a blinded inter-observer analysis of ten randomly selected patients was performed by two experienced echocardiographers. Continuous TTE inter-rater data were compared by calculating an intraclass correlation coefficient (ICC(A,3)) using the software R. In this statistical test, a significant p-value indicates agreement beyond what would be expected by chance.

## Results

### Establishing MAD + MVP and MVP-only groups and MAD subgroups

The *MAD + MVP* group (*N* = 41) was based on CMR findings alone. The total *MVP-only* group (*N* = 82) was comprised of both CMR-detected (*N* = 15) and TTE-detected MVP (*N* = 67) patients. The imbalance of group sizes reflects the way patients were identified in an advanced imaging cohort rather than the true prevalence of MAD among all patients with MVP. We compared the CMR and TTE-detected *MVP-only* patients to the TTE-detected *MVP-only* group to ensure there were no significant differences, confirming their comparability in demographics (sex, age) and echocardiographic parameters (Supplementary Table 1). Patients with *MAD + MVP* were then stratified into MAD < 5 mm (*N* = 13), MAD ≥ 5 mm (*N* = 28), and MAD ≥ 10 mm (*N* = 10) to elucidate further how increasing size and severity of MAD affects echocardiographic and CMR parameters.

### Echocardiographic findings

The degree of MAD assessed by TTE versus CMR did not differ significantly (7.0 mm vs. 7.8 mm, mean difference 0.8 mm, *p* = 0.269). Inter-observer variability in MAD measurements by echocardiography illustrated a strong agreement between observers (ICC = 0.897, *p* < 0.001). Similarly, end-systolic and end-diastolic annulus size measurements demonstrated a high level of agreement with ICCs of 0.861 and 0.809, respectively (*p* < 0.001 and *p* = 0.001).

Comparing patients with *MAD + MVP* (and MAD subgroups) to those with *MVP-only* revealed significant echocardiographic differences in annular dimensions (Table 1):

**Table 1:** Mean Echocardiographic Parameters

TTE parameters	MVP ONLY (*n* = 82)	ALL MAD (*n* = 41)	MAD < 5 mm (*n* = 13)	MAD ≥ 5 mm (*n* = 28)	MAD ≥ 10 mm (*n* = 10)
Mitral annular end-systolic dimension	31.2 (± 4.8)	33.2 (± 5.8)	28.6 (± 3.1) *	35.1 (± 5.7) †	38.1 (± 3.9) ‡
Mitral annular end-diastolic dimension	29.7 (± 5.2)	29.8 (± 5.2)	26.3 (± 2.7) †	31.4 (± 5.4)	32.3 (± 4.8)
Mitral annular change from diastole to systole	1.5 (± 4.0)	3.4 (± 3.5) †	2.3 (± 3.2)	3.8 (± 3.7) †	5.8 (± 3.2) †

1. The *MAD + MVP* group demonstrated a greater change in annulus size (mean difference 1.9 mm, *p* = 0.009).
2. The MAD < 5 mm group compared to MVP-only had a smaller end-systolic annular dimension (mean difference − 2.6 mm, *p* = 0.024) and smaller end-diastolic dimension (mean difference − 3.4 mm, *p* = 0.002).
3. For patients with MAD < 5 mm compared to MAD ≥ 5 mm, the annulus end-systolic size (mean difference − 6.5 mm, *p* < 0.001) and end-diastolic size (mean difference − 5.1 mm, *p* < 0.001) were significantly different.
4. Patients with MAD ≥5 mm compared to *MVP-onl*y had larger annulus end-systolic size (mean difference 3.9 mm, *p* = 0.002) and greater change in annulus size (mean difference 2.3 mm, *p* = 0.008).
5. The MAD ≥ 10 mm group showed an even larger annulus end-systolic size (mean difference 6.9 mm, *p* < 0.001) and a greater annulus size change (mean difference 4.3 mm, *p* = 0.002) than MVP-only.

**Fig. 3:**
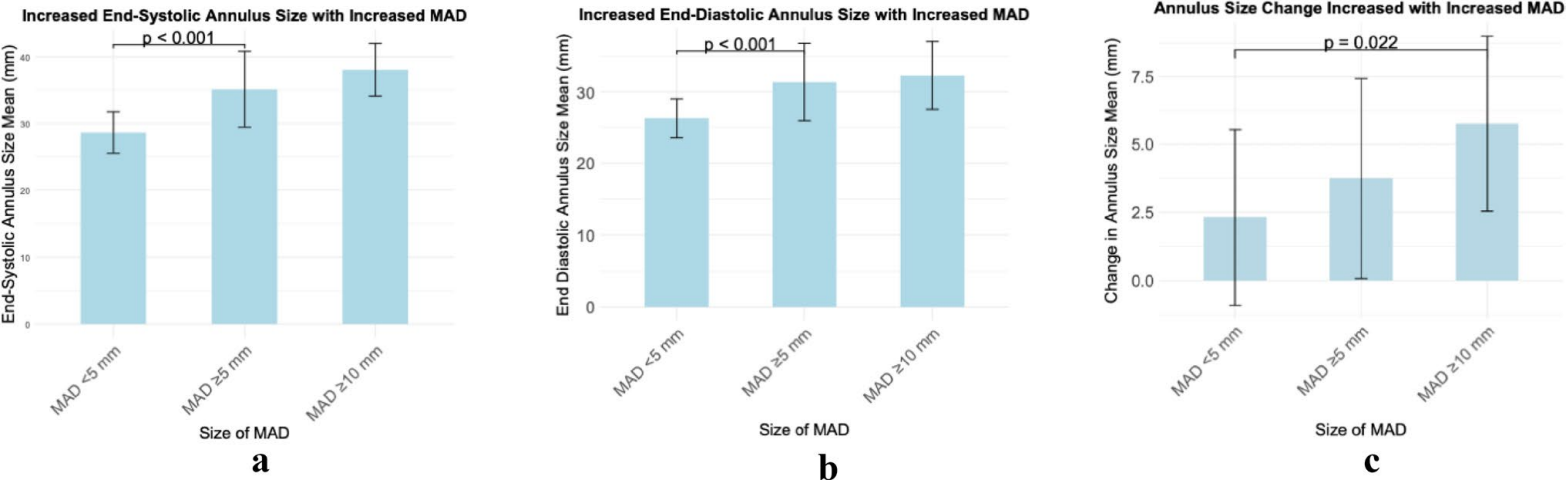
Mitral Annular Disjunction is Associated with Increased Annular Dimensions in Diastole andSystole, with Paradoxical Systolic Expansion

Thus, with increasing size of MAD, there was an increase in end-systolic (Fig. 3a), end-diastolic size (Fig. 3b), and difference between end-diastolic and end-systolic size (Fig. 3c). These changes were significant when comparing end-systolic and end-diastolic annulus size from MAD <5 mm to all other groups ≥5 mm, although the increase in annulus size was statistically significant only between MAD <5 mm and MAD ≥ 10 mm. Notably, when comparing any degree of *MAD + MVP* to *MVP-only*, there were no differences in end-systolic or end-diastolic dimensions. However, unlike patients with *MVP-only*, patients with any degree of MAD had a significantly larger end-systolic compared to end-diastolic annulus size (*p* = 0.007). There were also no significant differences in the presence of the Pickelhaube sign on echocardiography in patients with MAD compared to MVP.

### MRI findings

Comparing *MAD + MVP* (and MAD subgroups) to those with *MVP-only* revealed significant CMR differences in CO and CI (Table 2):

**Table 2:** Mean CMR Parameters

CMR parameters	MVP ONLY (*n* = 15)	ALL MAD (*n* = 41)	MAD < 5 mm (*n* = 13)	MAD ≥ 5 mm (*n* = 28)	MAD ≥ 10 mm (*n* = 10)
LVEDV (mL)	129.13 (± 65.74)	128.23 (± 33.97)	126.00 (± 36.94)	128.27 (± 33.54)	134.75 (± 27.67)
LVESV (mL)	63.86 (± 56.44)	55.42 (± 20.56)	54.52 (± 24.44)	55.19 (± 19.19)	54.63 (± 14.33)
LVEF (%)	59.46 (± 17.40)	51.63 (± 19.00)	50.59 (± 24.54)	52.12 (± 16.90)	51.07 (± 19.61)
RVEF (%)	50.63 (± 14.46)	47.19 (± 18.96)	44.26 (± 27.50)	48.50 (± 14.30)	50.60 (± 5.73)
SV (mL/beat)	71.43 (± 18.74)	72.64 (± 20.43)	72.25 (± 20.36)	72.69 (± 21.24)	78.75 (± 16.34)
CO (L/min)	5.47 (± 1.59)	4.57 (± 1.13)	4.80 (± 1.52)	4.48 (± 0.95) *	4.41 (± 0.80) *
CI (L/min/m^2^)	3.03 (± 0.64)	2.57 (± 0.68) *	2.55 (± 0.76)	2.59 (± 0.66) *	2.52 (± 0.44) *
Subjects with LGE (%)	33.33	48.78	41.67	53.57	60.00 *
Mitral regurgitant volume (mL/beat)	16.86 (± 20.11)	7.81 (± 12.32)	0.91(± 3.02) †	11.46 (± 13.78)	15.43 (± 15.26)
Mitral regurgitant fraction (%)	22.13 (± 32.21)	9.89 (± 14.16)	2.36 (± 7.89) *	14.10 (± 15.26)	19.86 (± 18.35)

**Fig. 4:**
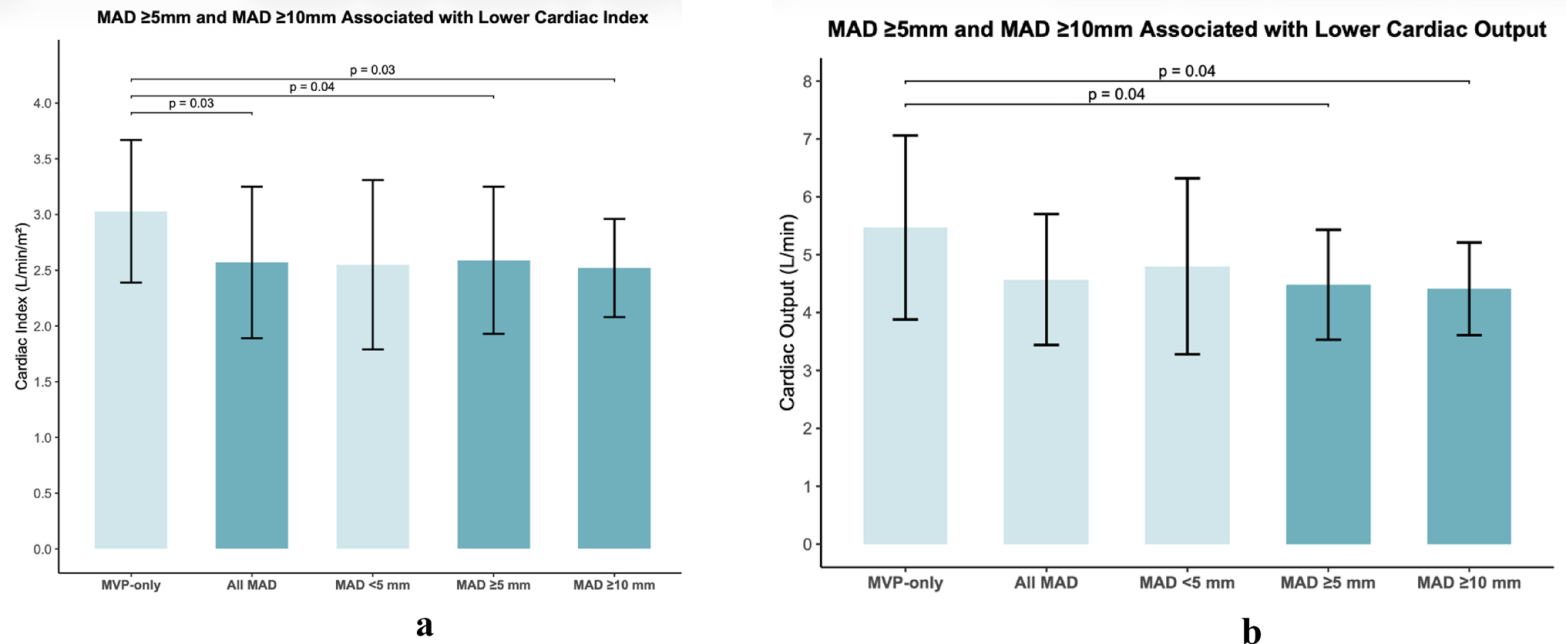
Mitral Annular Disjunction is Associated with Reduced Cardiac Output and Index

1. Patients with *MAD + MVP* demonstrated a lower CI as compared to the *MVP-only* group (mean difference − 0.47 L/min/m^2^, *p* = 0.026, Fig. 4a).
2. Patients with MAD ≥5 mm and MAD ≥ 10 mm had reduced CI (mean difference − 0.46 L/min/m^2^, *p* = 0.042 and mean difference − 0.51 L/min/m^2^, *p* = 0.027, respectively, Fig. 4a) and CO (mean difference − 0.99 L/min, *p* = 0.040 and mean difference − 1.06 L/min/m^2^, *p* = 0.039, Fig. 4b) as compared to *MVP-only*.
3. Patients with MAD ≥ 10 mm had a greater incidence of LGE as compared to those with MAD < 10 mm (*p* = 0.028).
4. There were no differences in the incidence of MR, regurgitant volume, or regurgitant fraction between the *MAD + MVP* and *MVP-only* groups, nor amongst the MAD subgroups (except <5 mm) as compared to MVP-only.
5. Patients with MAD <5 mm showed no significant differences in CMR parameters compared to *MVP-only*, aside from MR.
6. No significant differences were observed in LVEDV, LVESV, LVEF, RVEF, or regional wall motion abnormalities between any *MAD + MVP* group and the *MVP-only* group.

Despite the differences in CO and CI observed in the *MAD + MVP* and MAD ≥ 5 mm groups compared to *MVP-only*, ANOVA analysis of cardiac output and cardiac index illustrated no significant differences across MAD subgroups with p values of 0.203 and 0.110, respectively. Further, there were no differences in the incidence of MR, regurgitant volume, or regurgitant fraction between the *MAD + MVP* or MAD subgroups (except < 5 mm) and *MVP-only* groups.

### Comparison of MVP severity in MAD + MVP vs. MVP-only

We compared *MAD + MVP* (and its subgroups) to *MVP-only* in terms of severity as reported on TTE and CMR (Supplementary Table 2).

1. There was no significant difference in the burden of mild vs. moderate/severe MVP between any groups.
2. For patients with *MAD + MVP*, any degree of MAD (mean difference 1.8 mm, *p* = 0.002) and MAD ≥5 mm (mean difference 1.9 mm, *p* = 0.002) had significantly lower MVP height, compared to *MVP-only*, but there was no difference in MVP height between MAD ≥ 10 mm and *MVP-only*.

## Discussion

### MRI insights into disturbances in cardiac function in patients with MAD

Despite the well-established associations of MAD with myocardial fibrosis of the inferobasal wall of the ventricle and the papillary muscles [16, 17], the direct impact of MAD on cardiac function remains incompletely defined. While MAD is associated with annular and basal left ventricular enlargement, most studies have not demonstrated a significant reduction in global systolic function or cardiac index attributable to MAD alone [18, 19]. Strain imaging by both TTE and CMR reveals regional dysfunction in basal segments, but global measures such as ejection fraction and cardiac index are typically preserved unless there is advanced remodelling or significant mitral regurgitation [20]. The prognostic value of cardiac index is well established in heart failure populations [21], but its specific role in MAD without overt heart failure or severe regurgitation remains unclear.

Our study demonstrates, for the first time, that MAD, even at low levels, is associated with measurable adverse effects on cardiac function. Specifically, CMR imaging showed that MAD of any degree correlated with a reduction in CI, while MAD ≥ 5 mm was significantly associated with reductions in both CO and CI. Notably, these changes occurred independently of MR severity or the extent of MVP, indicating that the functional impairment is related to the mechanical effects of annular disjunction itself.

These findings of reduced CO and CI have not been demonstrated in prior studies, likely due to methodological differences. Much of the existing literature has focused on LVEF, LV size, MR severity, arrhythmias, or fibrosis, whereas our study specifically examined CO and CI–parameters that may detect more subtle functional impairment not apparent on LVEF alone. In our cohort, LVEF and LV dimensions were similar across groups, yet CI was reduced with any degree of MAD, and both CO and CI were significantly lower with MAD ≥5 mm. This suggests that MAD may be associated with early hemodynamic inefficiency rather than overt systolic dysfunction.

Additional factors may further explain these differences. Our analysis distinguished MVP-only from combined MAD and MVP and excluded pseudo-MAD, reducing potential misclassification. Moreover, while many studies utilise echocardiographic-derived measures; our study utilized phase-contrast CMRderived CO and CI, which may provide greater sensitivity for detecting subtle changes. Finally, prior studies often analyze MAD as a binary variable (present vs. absent) rather than evaluating across severity thresholds. In our cohort, MAD ≤ 5 mm–despite exceeding the conventional ≥ 2 mm definition– was not associated with changes in cardiac output or index. This aligns with prior literature suggesting that milder degrees of MAD (≤ 5 mm) may represent a normal variant. Inclusion of these cases in aggregate analyses of MAD could dilute pathologic changes in cardiac function and partially account for discrepancies across studies.

Further, myocardial fibrosis, as detected by LGE on CMR, is a key mediator of adverse outcomes in MVP and MAD [22, 23]. Fibrosis in the papillary muscles and inferobasal left ventricle is strongly associated with arrhythmic events and adverse prognosis, whereas MAD alone does not independently predict clinical outcomes in the absence of fibrosis [14, 16]. This underscores the importance of tissue characterization in risk stratification.

Our study demonstrated that patients with MAD ≥ 10 mm had significantly greater scar burden as measured by LGE on CMR. This was not observed in patients with lesser degrees of *MAD + MVP* or *MVP-only*, suggesting a threshold effect wherein extensive MAD drives progressive myocardial fibrosis.

The association between greater MAD severity and increased fibrosis likely reflects chronic mechanical stress imposed on the myocardium by annular separation and its abnormal motion. This mechanical tension, particularly on the basal inferolateral myocardium and papillary muscles, may trigger microtrauma, local inflammation, and subsequent fibrotic remodeling over time. The localization and presence of scarring in MAD ≥ 10 mm patients—despite no difference in MR burden—supports this hypothesis.

Fibrosis is a known substrate for ventricular arrhythmias and systolic dysfunction, raising important implications for long-term clinical outcomes in patients with significant MAD. While arrhythmic risk was not the focus of our study, the presence of fibrosis in these patients suggests the potential for a progressive disease course warranting further investigation and closer clinical surveillance.

### MAD as an independent pathological process

Importantly, our data indicate that the observed abnormalities in MAD patients cannot be attributed to more severe MVP or MR. MVP height and MR parameters were similar between *MAD + MVP* and *MVP-only* groups, and even lower in some MAD subgroups. We believe this is a novel observation, and reinforces the concept that MAD introduces independent mechanical pathology, with implications for both contractile performance and myocardial integrity.

These findings may explain the heterogeneity of clinical presentations among MVP patients and support a paradigm shift in which MAD is not merely a modifier of MVP, but a distinct clinical and imaging phenotype with independent risk potential.

### Echocardiographic support for MAD assessment

Although CMR was the primary tool for MAD identification in this study, transthoracic echocardiography (TTE) demonstrated high reliability in quantifying MAD and annular dimensions. Measurement agreement between TTE and CMR was excellent, and inter-observer variability was low. These results validate echocardiography as an accessible and reproducible screening tool in routine clinical practice, particularly when considering follow-up imaging or patient stratification based on MAD severity.

Additionally, patients with MAD exhibited greater systolic-diastolic annular size variation, with paradoxical annular expansion during systole—a finding consistent with earlier studies [24, 25]. This distinct annular motion pattern likely contributes to abnormal ventricular loading conditions and further supports the concept of MAD as a functional disruptor of mitral-ventricular coupling. Some prior studies [26, 27] have reported the Pickelhaube sign in association with MAD, but our data did not show a higher prevalence of this sign in MAD than in MVP-only.

## Limitations

While the gender demographics were similar across groups, the *MAD + MVP* group was significantly younger than the *MVP-only* group. Despite this, the *MAD + MVP* group exhibited poorer cardiac function and greater structural disturbances.

Sample sizes—particularly in the MAD ≥ 10 mm and *MVP-only* CMR cohorts—were modest, potentially limiting statistical power. We included TTE-detected MVP (with confirmed absence of MAD) to develop a larger *MVP-only* group, which could have introduced variability. However, no significant differences were found between *MVP-only* patients identified via CMR or TTE.

Our data indicate that TTE is a good screening tool for MAD that correlated with CMR. We acknowledge that there are other studies [28] that show CMR to be more sensitive in the detection of lesser degrees of MAD. We should state that a positive TTE is highly specific for predicting identification of MAD by CMR, but that TTE is not very sensitive and can miss lesser degrees of MAD that CMR *will* detect.

Further, this was a retrospective analysis, and the findings are limited by this fact. Finally, we did not correlate our findings with clinical outcomes.

## Conclusions

This study reinforces MAD as an independent pathological entity with functional and structural consequences distinct from MVP. The main conclusions are:

1. **MAD is associated with impaired cardiac performance**: CMR findings showed that MAD of any degree correlates with reduced CI, and MAD ≥ 5 mm and MAD ≥ 10 mm are linked to reductions in both CO and CI. These impairments were not explained by differences in mitral regurgitation severity or MVP extent, suggesting a direct effect of MAD on ventricular mechanics.
2. **Severe MAD is linked to myocardial fibrosis**: Patients with MAD ≥ 10 mm exhibited significantly higher scar burden on CMR compared to MVP-only and lesser degrees of MAD. This supports the hypothesis that chronic mechanical stress from progressive MAD contributes to myocardial remodeling and fibrosis.
3. **TTE is a reliable screening tool for MAD**: Transthoracic echocardiography demonstrated excellent agreement with CMR in detecting and quantifying MAD, with minimal inter-observer variability—even when including a trained medical student. These findings support the routine use of echocardiography for initial assessment and monitoring.
4. End-systolic mitral annular dimensions are larger in patients with MAD than in MVP-only and increase with increasing extent of MAD, although LVEF and ventricular dimensions are comparable.

Future research will be required to investigate the clinical impact of these electromechanical observations and determine how they affect long-term outcomes in MAD patients.

### Funding statement

No funding was received for this study.

**Declarations**:

### Conflict of interest

The authors declare that there are no conflicts of interest.

### Ethics approval

**statement**: This study was approved by the Institutional Review Board (IRB).

### Patient consent statement

Patient consent was not required for this study.

### Permission to reproduce material from other sources

Permission to reproduce material from other sources was not required.

## Supplementary Material

Below is the link to the electronic supplementary material.

Supplementary Material 1

## Data Availability

The data supporting this study’s findings are available from the corresponding author upon reasonable request.

## Declarations

### Permission to reproduce material from other sources

Permission to reproduce material from other sources was not required.
