# Supplementary material for: Mitral annular disjunction and mitral valve prolapse: echocardiographic-MRI insights into cardiac function
Source: Int J Cardiovasc Imaging. 2026 Apr 27;42(8):1591–600. doi: 10.1007/s10554-026-03709-6 (PMC13433552; doi:10.1007/s10554-026-03709-6)
Supplement: Supplementary file 1 [file 10554_2026_3709_MOESM1_ESM.docx]

# Supplementary Data:

**Supplementary Table 1: Comparison TTE-Detected MVP and CMR-Detected MVP Merged to Form the Combined MVP-only Control Group.**

| Variable | TTE- Detected MVP-Only (N=67) | CMR-Detected MVP-Only (N=15) | P Value | |
| --- | --- | --- | --- | --- |
| Age | 64 | 65 | 0.873 | |
| Female (%) | 64 | 47 | 0.209 | |
| End-Systolic Annulus Size (mm) | 31.33 (±4.57) | 30.59 (±5.83) | 0.650 | |
| End-Diastolic Annulus Size (mm) | 30.00 (±5.25) | 28.41 (±4.66) | 0.257 | |
| Annulus Size Change (mm) | 1.33 (±4.20) | 2.18 (±3.29) | 0.396 | |
| Data format: mean (±standard deviation) | | | |  |

**Supplementary Table 2: MVP Severity and Height**

| MVP Parameters | MVP ONLY (n=82) | ALL MAD (n=41) | MAD <5mm (n=13) | MAD ≥ 5mm (n=28) | MAD ≥ 10mm (n=10) |
| --- | --- | --- | --- | --- | --- |
| Mild MVP (%) | 75.61 | 90.25 | 100 | 89.29 | 80 |
| Moderate or Severe MVP (%) | 24.39 | 9.75 | 0 | 10.71 | 20 |
| MVP Height (mm) | 8.64 (±3.77) | 6.86 (±2.61) † | None Measured | 6.75 (±2.25) † | 8.10 (±3.40) |
| * p <0.05, † p <0.01, p<0.001 ‡. Data format: mean (±standard deviation) | | | | | |
